# Supplementary material for: The translocation activity of Rad54 reduces crossover outcomes during homologous recombination
Source: Nucleic Acids Res. 2024 Jun 3;52(12):7031–48. doi: 10.1093/nar/gkae474 (PMC11229335; doi:10.1093/nar/gkae474)
Supplement: gkae474_Supplemental_Files [file gkae474_supplemental_files.zip › Sridalla.etal_Supplement_F_New.pdf]

Supplemental Figure 1

A

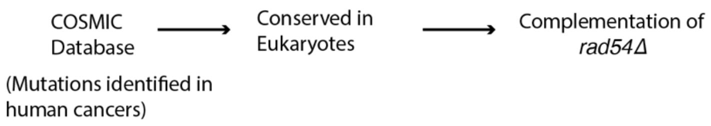

B

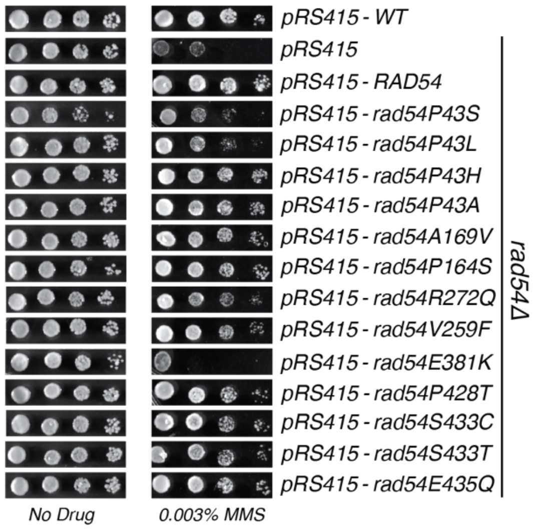

Supplemental Figure 1: Identification of Rad54 reduction of function allele
(A). An experimental setup is used to identify residues for testing. Amino Acid substitutions were identified in human cancers and tested to determine whether they were conserved in eukaryotes.
(B). Serial dilution yeast spot assay was used to test the complementation of *rad54*Δ sensitivity to the DNA alkylating agent MMS.

Supplemental Figure 2

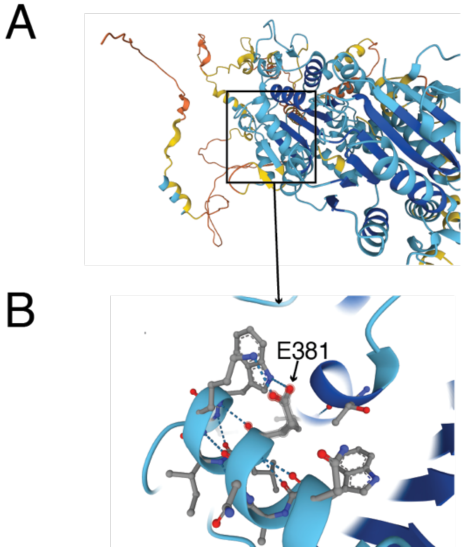

Supplemental Figure 2: Rad54E381K likely destabilizes an essential helix
(A). Alphafold2 structure of yeast Rad54. The alpha helix that contains the E381 residue is enclosed in a box. **(B).** Zoomed in view of the E381 residue. The residue likely contacts a Lysine and Histidine that stabilizes the helix. Conversion of the residue to Lysine disrupts the interaction and destroys the helix.

Supplemental Figure 3

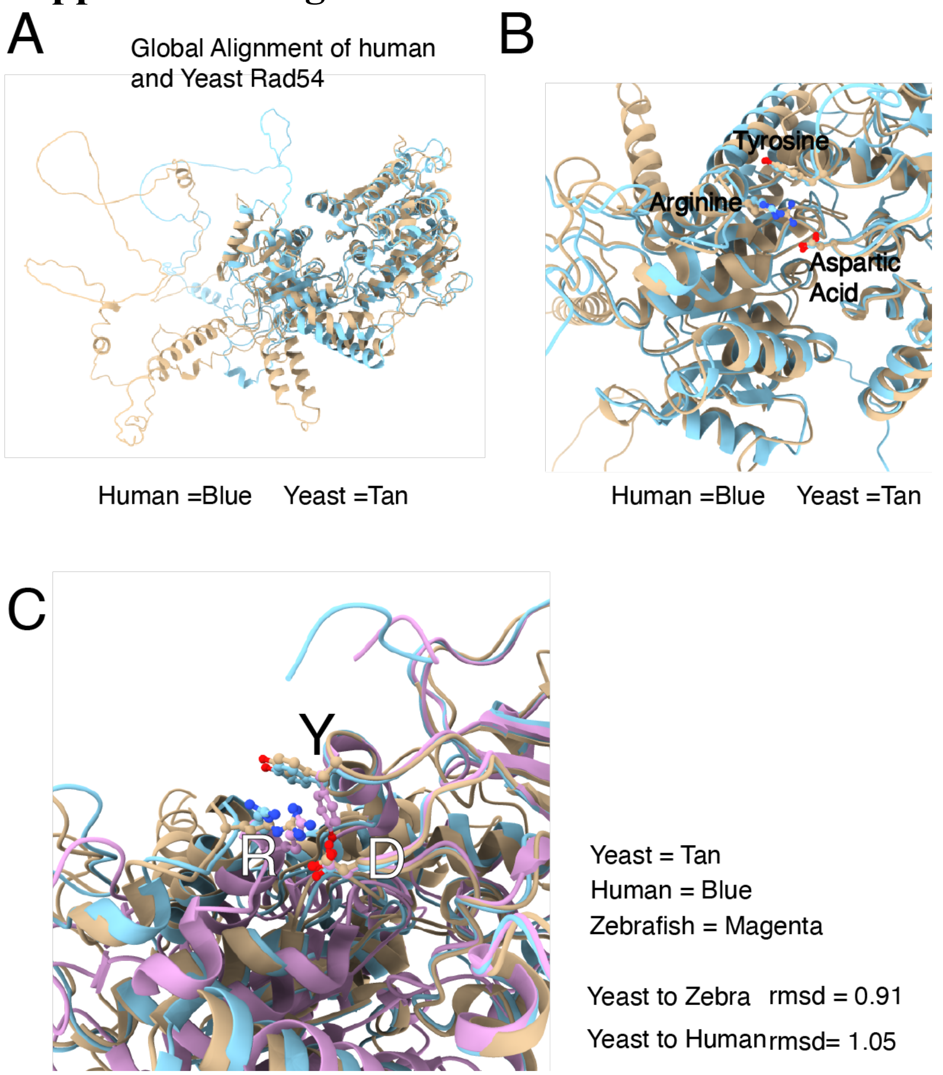

Supplemental Figure 3: Structural Overlay of Yeast, Human, and Zebrafish Rad54
(A). Structural superposition of human RAD54 (Blue) and yeast Rad54 (Tan) from alphafold2 generated structures of these molecules. The RMSD is 1.05. **(B).** Zoomed in structural overlay of human RAD54 and yeast Rad54. The latch domain residues are highlighted and in the same position in both structures. **(C).** Structural superposition of Human (alphafold2) blue, Yeast (alphafold2) tan, and Zebrafish (cystal structure PDB: 1Z3I) magenta.

Supplemental Figure 4

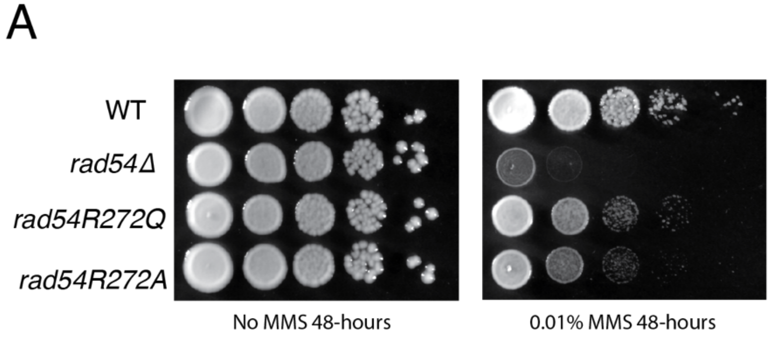

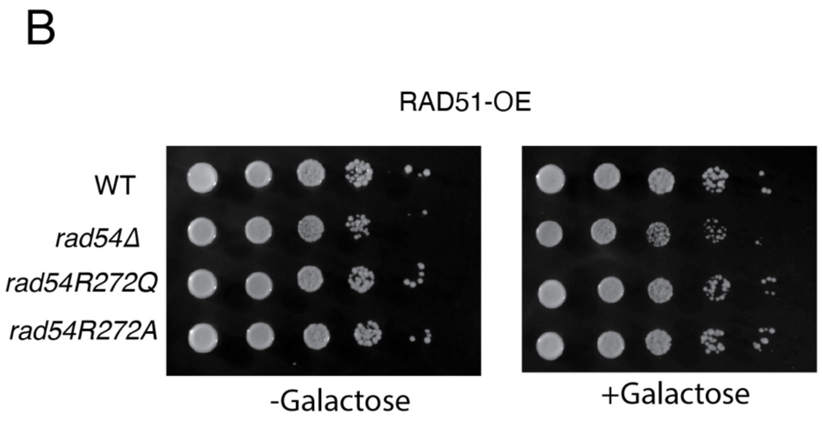

Supplemental Figure 4: Rad54 mutants complement the Rad51 overexpression phenotype (A). Serial dilution spot assay of WT, *rad54*Δ*, rad54R272Q, or rad54R272A* mutants spotted on YPD +/- 0.01% MMS. The spots represent a ten-fold dilution from the previous spot. **(B).** Serial dilution spot assay of WT, *rad54*Δ, *rad54R272Q,* or *rad54R272A* spotted on plates with or without galactose. The spots represent ten-fold dilutions from the previous spot.

Supplemental Figure 5

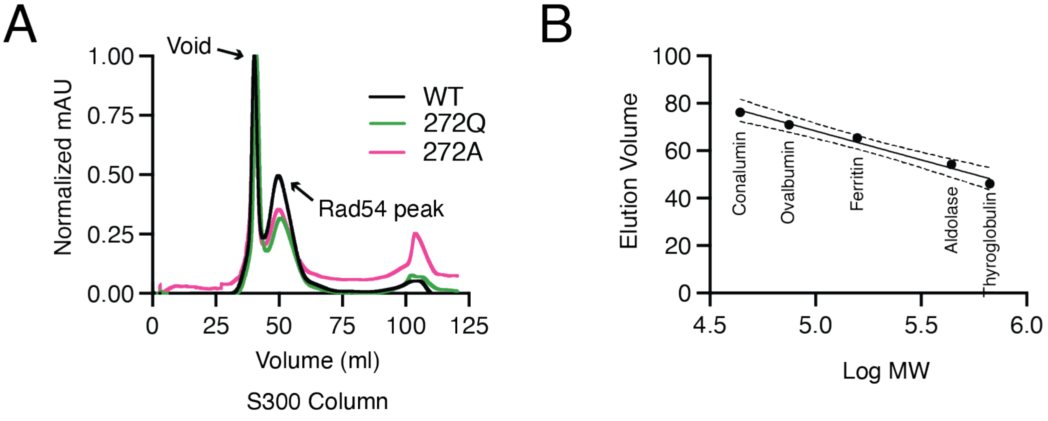

C

Elution Volume	log(mw)	Est. Number of Molecules	
49.6	5.768	3.79	WT
50.5	5.769	3.79	272Q
49.8	5.761	3.72	272A

Supplemental Figure 5: Rad54R272Q and Rad54R272A have the same oligomerization state as WT-Rad54
(A). Normalized chromatograph illustrating the elution profile of WT-Rad54 (black), Rad54R272Q (green), and Rad54R272A (magenta) from an S300 column. **(B).** Molecular weight standard for S300 column. The molecular weights (MW) log for several proteins is fit by linear regression (solid line). The dashed lines represent the 95% confidence interval for the fit. **(C).** A table illustrates the measured elution volume of WT-Rad54, Rad54R272Q, and Rad54R272A and estimates the number of molecules in the peak.

Supplemental Figure 6

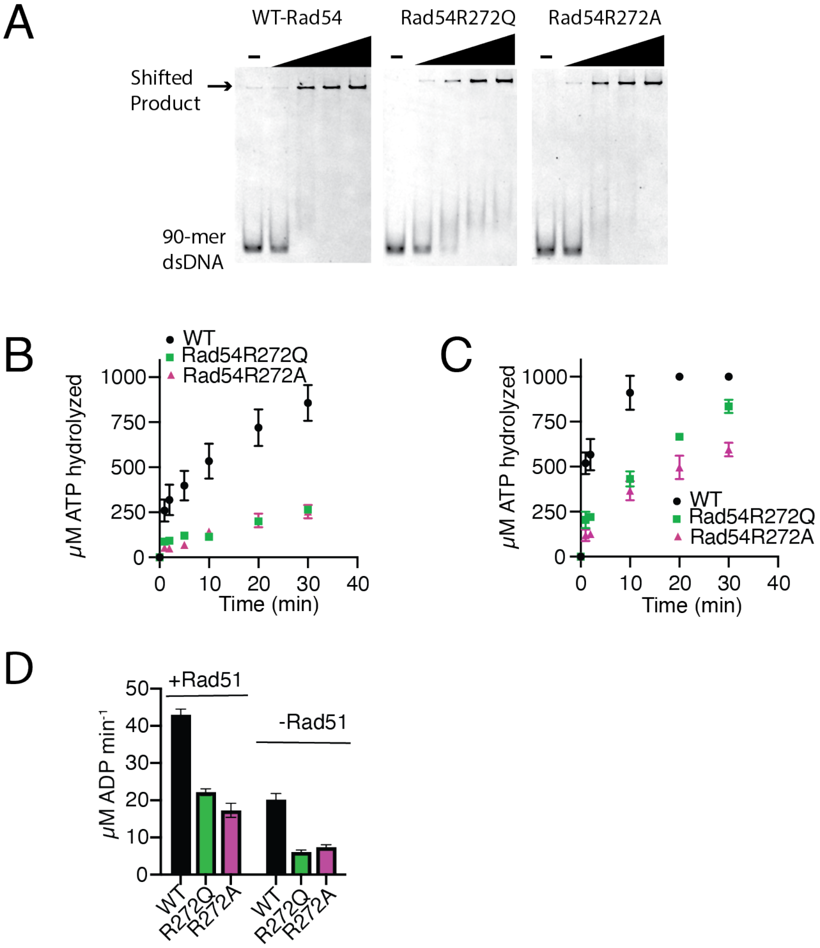

Supplemental Figure 6: Rad54 mutants are active for ATP hydrolysis with and without Rad51

(A). Electromobility shift assay (EMSA) for Rad54 binding to dsDNA. The WT-Rad54, Rad54R272Q, and Rad54R272A concentrations are 0, 6.25, 12.5, 25 and 50 nM. **(B).** ATP hydrolyzed (μM) by WT-Rad54 (Black), Rad54R272Q (Green), and Rad54R272A (Magenta) without Rad51 as a function of reaction time. The error bars represent the standard deviation of three independent experiments. **(C).** ATP hydrolyzed (μM) by WT-Rad54 (Black), Rad54R272Q (Green), and Rad54R272A (Magenta) with Rad51 as a function of reaction time. The error bars represent the standard deviation of three independent experiments. **(D).** The rate of ATP hydrolysis (μM ADP formed min^{-1}) was measured from the linear portion of the graphs in B and C for WT-Rad54 (Black), Rad54R272Q (Green), and Rad54R272A (Magenta). The error bars represent the confidence in the fit of the data.

Supplemental Figure 7

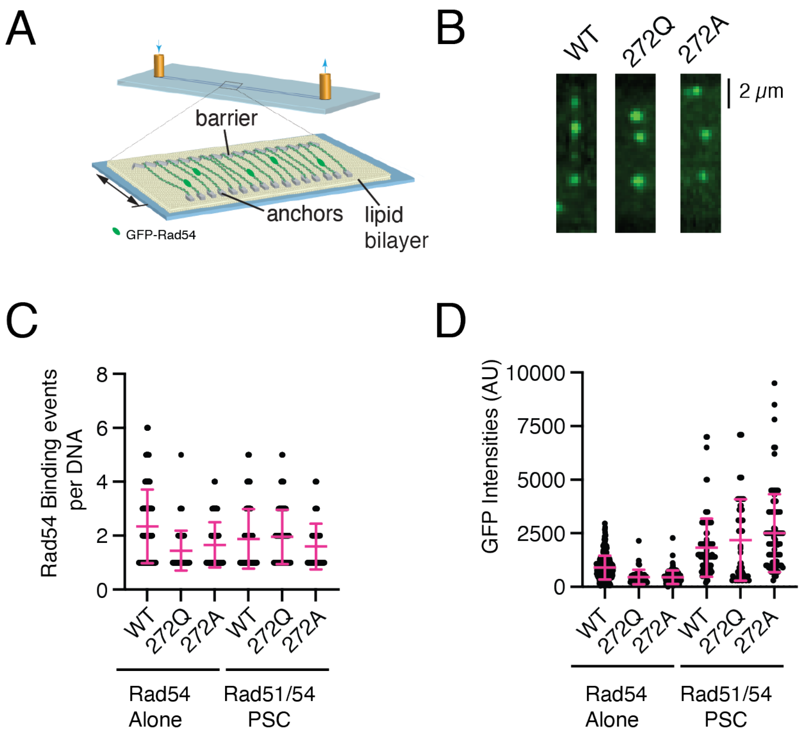

Supplemental Figure 7: Rad54R272Q and Rad54R272A interact with dsDNA
(A). Schematic cartoon illustrating the DNA curtains set-up to test Rad54 motor function dsDNA. **(B).** Widefield TIRFM microscope image of GFP-Rad54, GFP-Rad54R272Q, and GFP-Rad54R272A bound to dsDNA. **(C).** A quantification of Rad54 binding events for WT-Rad54, Rad54R272Q, and Rad54R272A alone or as part of the Rad51-PSC per DNA molecule. The bars represent the mean, and the error bars represent the standard deviation of the experiment. **(D).** A quantification of GFP intensities for WT-Rad54, Rad54R272Q, and Rad54R272A alone or as part of the Rad51-PSC. The bars represent the mean, and the error bars represent the standard deviation of the data.

Supplemental Figure 8

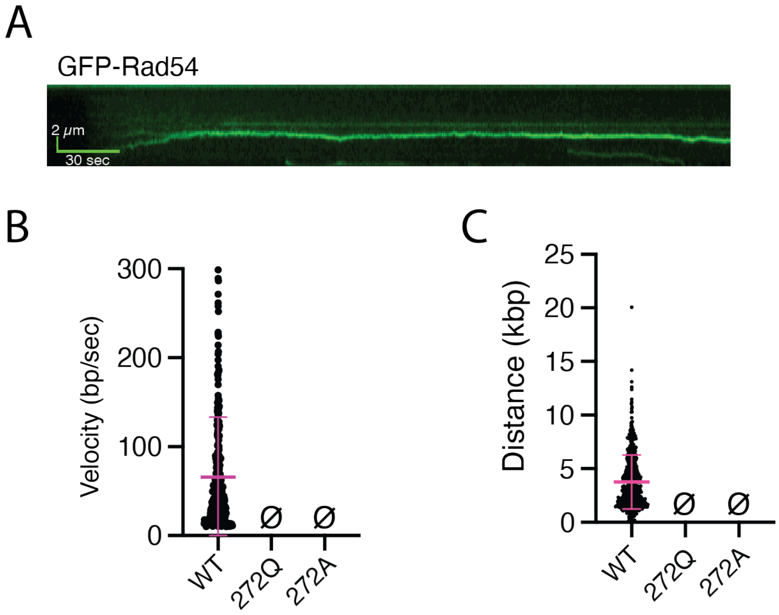

Supplemental Figure 8: Rad54R272Q and Rad54R272A do not show translocation activity in the absence of Rad51

A). Representative kymograph illustrating movement of WT-Rad54 along dsDNA. **(B).** The dot plot represents the velocities (N=278) for WT-Rad54, Rad54R272Q, and Rad54R272A. The crossbar represents the mean of the data, and the error bars represent the standard deviation of the data. **(C).** The dot plot represents the distance traveled (N=584) for WT-Rad54, Rad54R272Q, and Rad54R272A. The crossbar represents the mean of the data, and the error bars represent the standard deviation of the data.

Supplemental Figure 9

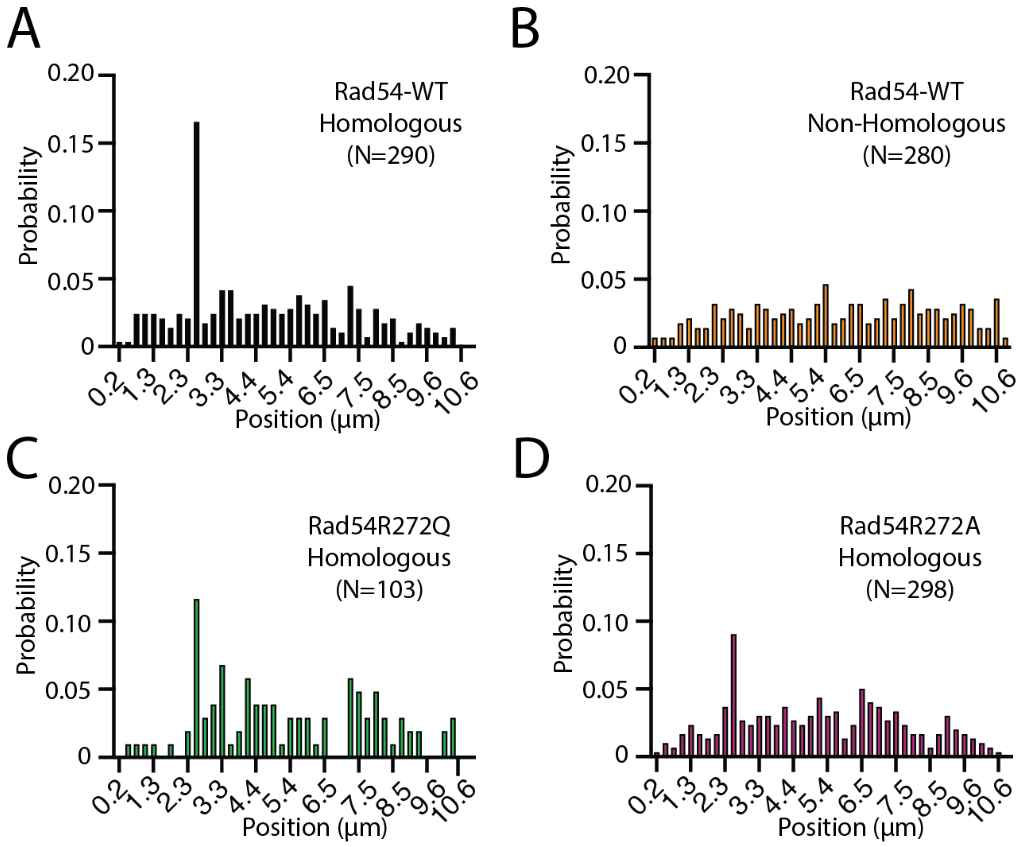

Supplemental Figure 9: Rad54R272Q and Rad54R272A have a reduction in sequence alignment

(A). Distribution of binding site probability for WT with homologous ssDNA (N=290). The data are also shown in Figure 4BC. **(B).** Distribution of critical site probability for WT-Rad54 with non-homologous ssDNA (N=290). The data are also presented in Figure 3BC. **(C).** Distribution of critical site probability for Rad54R272Q with homologous ssDNA (N=103). The data are also shown in Figure 4BC. **(D).** Distribution of binding site probability for Rad54R272A with homologous ssDNA (N=298). The data are also presented in Figure 4BC.

Supplemental Figure 10

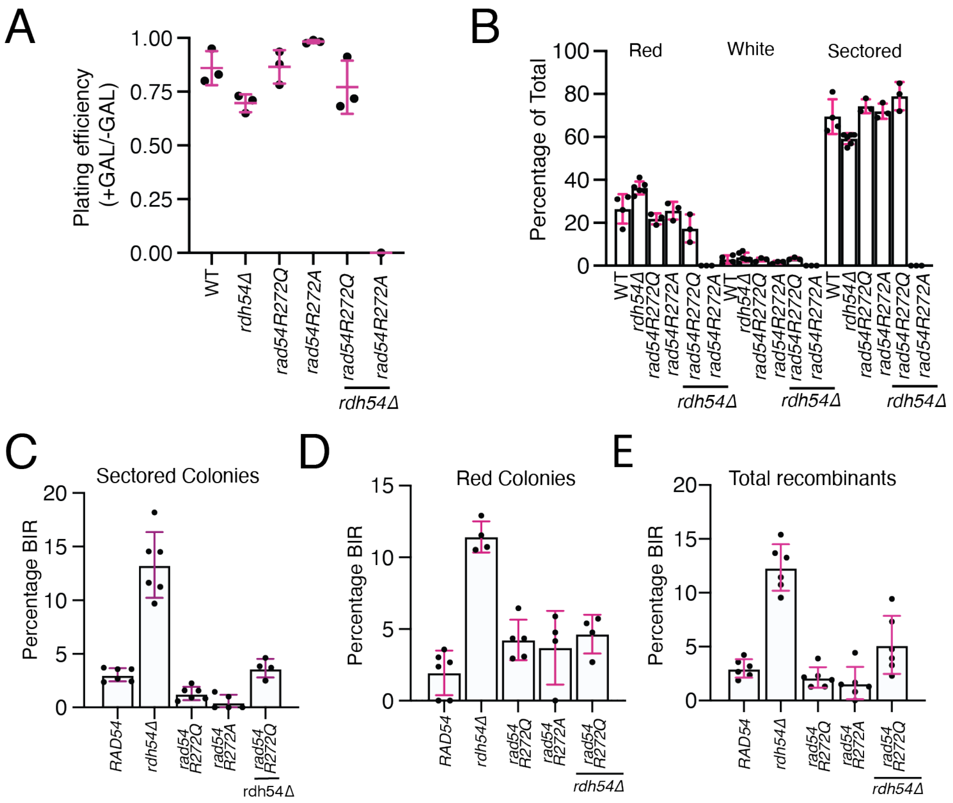

Supplemental Figure 10: Rad54 mutants result in altered BIR outcomes

(A). Measured plating efficiencies for WT, *rdh54*Δ, *rad54R272Q*, *rad54R272A*, *rad54R272Q rdh54*Δ, and *rad54R272A rdh54*Δ strains comparing survival with and without treatment with galactose. The Bar and error bars represent the mean and standard deviation of three independent experiments **(B).** Measured outcomes for gene conversion tracts as observed for red, white, and sectored colonies. Strains assayed include WT, *rdh54*Δ, *rad54R272Q*, *rad54R272A*, *rad54R272Q rdh54*Δ, and *rad54R272A rdh54*Δ. **(C).** Graph comparing the percentage of BIR outcomes in sectored colonies only for WT, *rdh54*Δ*, rad54R272Q, rad54R272A, rad54R272Q rdh54*Δ*, and rad54R272A rdh54*Δ. The error bars represent the standard deviation for at least three independent experiments **(D).** Graph comparing the percentage of BIR-like outcomes in solid red colonies only for WT, *rdh54*Δ*, rad54R272Q, rad54R272A, rad54R272Q rdh54*Δ*, and rad54R272A rdh54*Δ. The error bars represent the standard deviation for at least three independent experiments **(E).** Graph representing the prevalence of BIR-like events in total recombinants for WT, *rdh54*Δ*, rad54R272Q, rad54R272A, rad54R272Q rdh54*Δ*, and rad54R272A rdh54*Δ.The error bars represent the standard deviation of at least three independent experiments

Supplemental Information
Supplemental Table 1

Strains	Genotype	Source or reference
LSY-2202-15D (WT)	*MATa ade2-n his3::NatMX4 met22:klURA3*	*(Mazon et al. 2010)*
LSY-2205-11C (WT)	*MATalpha ade2-I lys2:GAL-ISCE1 his3:HphMX4*	*(Mazon et al. 2010)*
BY4741 (WT)		*Dharmacon*
BY4741	*rad54Δ*	*Dharmacon*
BY4741	*RAD54::rad54R272Q*	*This study*
BY4741	*RAD54::rad54R272A*	*This study*
JBC0105	*15D-RAD54-KANMX*	*This study*
JBC0106	*11C-RAD54-KANMX*	*This study*
JBC0107	*15D-rad54R272Q-KANMX*	*This study*
JBC0108	*11C-rad54R272Q-KANMX*	*This study*
JBC0109	*15D-rad54R272A-KANMX*	*This study*
JBC0110	*11C-rad54R272A-KANMX*	*This study*
JBC0241	*15D-RAD54-KANMX rdh54Δ::HIS3*	*This study*
JBC0236	*11C-RAD54-KANMX rdh54Δ::HIS3*	*This study*
JBC0243	*15D-rad54R272Q-KANMX rdh54Δ::HIS3*	*This study*
JBC0238	*11C-rad54R272Q-KANMX rdh54Δ::HIS3*	*This study*
JBC0245	*15D-rad54R272A-KANMX rdh54Δ::HIS3*	*This study*
JBC0240	*11C-rad54R272A-KANMX rdh54Δ::HIS3*	*This study*
JBC0210	*S. cerevisiae protease deficient strain*	
Diploids		*This study*
JBC0105XJBC0106	*RAD54-KANMX/RAD54-KANMX*	*This study*
JBC0107XJBC0108	*rad54R272Q/rad54R272Q*	*This study*
JBC0109XJBC0110	*rad54R272A/rad54R272A*	*This study*
JBC0241XJBC0236	*RAD54-KANMX/RAD54-KANMX rdh54Δ/rdh54Δ*	*This study*
JBC243XJBC248	*rad54R272Q/rad54R272Q rdh54Δ/rdh54Δ*	*This study*
JBC0245XJBC0240	*rad54R272A/rad54R272A rdh54Δ/rdh54Δ*	*This study*

Supplemental Table 1: Strain list of all strains used in this study

Supplemental Table 2

Name	Source
pRS415	
pRS415-RAD54	(Crickard et al., 2020)
pRS415-rad54P43S	This study
pRS415-rad54P43L	This study
pRS415-rad54P43H	This study
pRS415-rad54P43A	This study
pRS415-rad54A169V	This study
pRS415-rad54P164S	This study
pRS415-rad54R272Q	This study
pRS415-rad54V259F	This study
pRS415-rad54E381K	This study
pRS415-rad54P428T	This study
pRS415-rad54S433C	This study
pRS415-rad54S433T	This study
pRS415-rad54E435Q	This study
pRS415-rad54R272A	This study
pRS415-rad54Y562A	This study
pRS415-rad54D769A	This study
pRS415-rad54D769H	This study
pRS305-*RAD54-KANMX*	This study
pRS305-*rad54R272Q-KANMX*	This study
pRS305-*rad54R272A-KANMX*	This study
pYES-GST-RAD54	
pYES-GST-GFP-RAD54	Crickard et al. 2018
pYES-GST-rad54R272Q	This study
pYES-GST-GFP-rad54R272Q	This study
pYES-GST-rad54R272A	This study
pYES-GST-GFP-rad54R272A	This study
pYES-RAD51	
pET11C-SUMO-yRad51	Crickard et al. 2020
P11D-sctRPA	
P11D-sctRPA-mCherry-70	
pUC19 plasmid	New England Biolabs

Supplemental Table 2: List of all the plasmids used in this study

Supplemental Table 3

hRAD54L mutation	yRad54 residue
P50S	43
P50L	43
P50H	43
P50A	43
P112S	164
A117V	169
V141F	259
R154Q	272
E229K	381
P269T	428
S274C	433
S274T	433
E276Q	435
D610H*	769

*The D610H mutation was also found in COSMIC, but the respective yeast mutant *rad54D769H* was not created for the initial complementation spot assay. This mutant was created for the targeted complementation spot assay after it was found that residue D769 is involved in a stabilizing interaction.

Supplemental Table 3: Comparison of human and yeast amino acid positions in Rad54 tested in this study

Supplemental Table 4
Gene Conversion outcomes

Strain	Solid Red	Solid White	Sectored	Total
WT	249	26	707	979
rad54R272Q/rad54R272Q	226	15	667	908
rad54R272A/rad54R272A	131	15	595	741
rdh54Δ/rdh54Δ	183	17	443	644
rad54R272Q/rad54R272Q rdh54Δ	178	19	291	488
rad54R272A/rad54R272A rdh54Δ	0	0	0	0

Supplemental Table 5
Sectored

Strain	CO	NCO	BIR	Total	Chromosome Loss
WT	356	329	22	707	0
rad54R272Q/rad54R272Q	401	260	6	667	0
rad54R272A/rad54R272A	354	237	4	595	3
rdh54Δ/rdh54Δ	202	187	54	443	0
rad54R272Q/rad54R272Q rdh54Δ	224	52	15	291	5
rad54R272A/rad54R272A rdh54Δ	0	0	0	0	N/A

Supplemental Table 6
Solid Red

Strain	CO	NCO	BIR	Total
WT	34	208	7	249
rad54R272Q/rad54R272Q	44	175	7	226
rad54R272A/rad54R272A	46	123	5	131
rdh54Δ/rdh54Δ	20	146	17	183
rad54R272Q/rad54R272Q rdh54Δ	46	124	8	178
rad54R272A/rad54R272A rdh54Δ	0	0	0	0

Supplemental Table 7

Name	Sequence	Homologous region
Labeling Oligo	Atto647N-CCGCCTCGCAGAACGGGCATTCCCT	N/A
65-mer pUC19 homology	AGGGAATGCCCGTTCTGCGAGGCGGTGGATCTCAACAGCGGTAAGA<br>TCCTTGAGAGTTTTCGCCCCGAAGAACGTTTTCCAATGATGAGC	2348-<br>2373
90-mer pUC19 homology	AGGGAATGCCCGTTCTGCGAGGCGGGGTGCACGAGTGGGTTACATCG<br>AACTGGATCTCAACAGCGGTAAGATCCTTGAGAGTTTTCGCCCCG<br>AAGAACGTTTTCCAATGATGAGC	2283-<br>2373
130-mer pUC19 homology	AGGGAATGCCCGTTCTGCGAGGCGGGGTGCACGAGTGGGTTACATCG<br>AACTGGATCTCAACAGCGGTAAGATCCTTGAGAGTTTTCGCCCCGAAG<br>AACGTTTTCCAATGATGAGCACTTTTAAAGTTCTGCTATGTGGCGCGGT<br>ATTATCCCGTA	2243-<br>2373
90-mer Lambda homology	Atto647N-GATGTTCTGCTGGATATGCACTTTTCCGGGC<br>TGACGTACACCGTGCTCAGCCTGTTTTTCA<br>GCGATCCGGATATGCATCCGCTGGATTTC	10,253-<br>10,342
90-mer Lambda		N/A
EMSA<br>Unlabeled strand	GACCATGATTACGCCAAGCTTGCATGCCTGCAGGTCGACTCTAGAGGATC<br>CCCGGGTACCGAGCTCGAATTCACTGGCCGTCGTTTTACA	
90-mer-fluor EMSA	Atto647N-<br>TGTAAAACGACGGCCAGTGAATTCGAGCTCGGTACCCGGGGATCCTCTAG<br>AGTCGACCTGCAGGCATGCAAGCTTGGCGTAATCATGGTC	

Supplemental Table 7: DNA sequences used for homology search and D-loop experiments
